# Reproducibilidad y validez de criterio de dos técnicas radiográficas para variaciones de premolares mandibulares comparadas con CBCT

**DOI:** 10.15649/cuidarte.2300

**Published:** 2022-08-28

**Authors:** Martha Liliana Rincón Rodriguez, Ruth Aralí Martínez Vega, Martha Lucely Duarte, Jaime O Moreno

**Affiliations:** 1 Universidad Santo Tomás (USTA), Bucaramanga, Colombia. Email: martha.rincon@ustabuca.edu.co Universidad Santo Tomás Universidad Santo Tomás Bucaramanga Colombia martha.rincon@ustabuca.edu.co; 2 Universidad de Santander (UDES), Bucaramanga, Colombia. Email: rutharam@yahoo.com Universidad de Santander Universidad de Santander Bucaramanga Colombia rutharam@yahoo.com; 3 Universidad Santo Tomás (USTA), Bucaramanga, Colombia. Email: martha.duarte@ustabuca.edu.co Universidad Santo Tomás Universidad Santo Tomás Bucaramanga Colombia martha.duarte@ustabuca.edu.co; 4 Universidad Santo Tomás (USTA), Bucaramanga, Colombia. Email: jaime.moreno@ustabuca.edu.co Universidad Santo Tomás Universidad Santo Tomás Bucaramanga Colombia jaime.moreno@ustabuca.edu.co

**Keywords:** Reproducibilidad, Validez, Diente Premolar, Reproducibility, Validity, Bicuspid, Reprodutibilidade, Validade, Dente Pré-Molar

## Abstract

**Introducción:**

La existencia de variaciones anatómicas ocasiona fracasos en tratamientos endodónticos, por lo que es importante diagnosticarlas. El objetivo fue determinar la reproducibilidad y la validez de criterio de las radiografías con placa de fósforo y la radiovisografía con sensor para identificar las variaciones anatómicas detectadas por tomografía computarizada de haz cónico (CBCT) en premolares inferiores.

**Métodos:**

En 140 premolares se obtuvieron imágenes por CBCT, radiografía y radiovisografía. Se realizó lectura independiente por dos endodocistas, evaluándose la clasificación de Vertucci y las ramificaciones. Se determinó la reproducibilidad intraobservador e interobservador. Se calcularon sensibilidad, especificidad y áreas bajo la curva operador-receptor (AUC) utilizando como estándar de oro la CBCT.

**Resultados:**

La reproducibilidad intraobservador e interobservador fue mayor para radiografía. Para la Clase I de Vertucci, la radiografía presentó mayor sensibilidad (94,7%), especificidad (64,9%) y AUC (0,795) que la radiovisiografía (89,3%, 62,2% y 0,757, respectivamente), al igual que para la Clase V (Radiografía 69,2%, 93% y 0,811; Radiovisiografía 50%, 84,2% y 0,671, respectivamente). Ninguna de las técnicas aportó al diagnóstico de la Clase III (AUC <0,5). Las ramificaciones fueron infrecuentes (2,9%) y su detección fue baja (Sensibilidad 25% para radiografía y 0% para radiovisiografía).

**Discusión:**

Este es el primer estudio que evalúa la reproducibilidad y validez de estas dos técnicas radiográficas comparadas con la CBCT para la detección de variaciones anatómicas en dientes.

**Conclusiones:**

La radiografía con placa de fósforo presentó mayor reproducibilidad y validez para el diagnóstico de las Clase I y V de Vertucci, que fueron las variaciones más frecuentes.

## Abstract (en)

**Introduction:**

Considering that the existence of anatomical variations causes endodontic treatment failures, therefore it is important to diagnose them. This study aimed to determine the reproducibility and criterion validity of phosphor plate radiographs and sensor-based radiovisiography to identify anatomical variations detected by cone-beam computed tomography (CBCT) in lower premolars.

**Materials and Methods:**

140 premolars images were obtained by CBCT, radiographs and radiovisiography. Independent interpretation was performed by two endodontists to evaluate the Vertucci classification and ramifications. Intraobserver and interobserver reproducibility were determined. Sensitivity, specificity and areas under the receiver-operator curve (AUC) were calculated using CBCT as the gold standard.

**Results:**

Intraobserver and interobserver reproducibility was higher for radiography. For Vertucci type I, radiography showed higher sensitivity (94.7%), specificity (64.9%) and AUC (0.795) than radiovisiography (89.3%, 62.2% and 0.757, respectively), similarly for type V (radiography at 69.2%, 93% and 0.8111; radiovisiography at 50%, 84.2% and 0.671, respectively). None of the techniques contributed to the diagnosis of type III (AUC < 0.5). Ramifications were infrequent (2.9%) with a low level of detection (sensitivity at 25% for radiography and 0% for radiovisiography).

**Discussion:**

This is the first study to evaluate the reproducibility and validity of these two radiographic techniques compared with CBCT for the detection of anatomical variations in teeth.

**Conclusions:**

Phosphor plate radiography showed higher reproducibility and validity for the diagnosis of Vertucci types I and V, which were the most frequent premolar variations found. This is a dissertation for the Master’s degree in Dentistry available in the repository of the Universidad Santo Tomas, Bucaramanga campus.

## Resumo

**Introdução:**

A existência de variações anatômicas causa falhas no tratamento endodôntico, por isso é importante diagnosticá-las. O objetivo foi determinar a reprodutibilidade e validade dos critérios das radiografias em placas de fósforo e radiovisiografia sensorial para identificar variações anatômicas detectadas pela tomografia computadorizada de feixe cônico (TCFC) em pré-molares inferiores.

**Métodos:**

obtiveram-se TCFC, imagens radiográficas e radiovisográficas em 140 pré-molares. A leitura independente foi realizada por dois endodontistas, avaliando a classificação Vertucci e suas ramificações. Foi determinada a reprodutibilidade intra e interobservador. Sensibilidade, especificidade e áreas sob a curva operação do receptor (AUC) foram calculadas utilizando a TCFC como padrão-ouro.

**Resultados:**

A reprodutibilidade intra e inter-observador foi maior para a radiografia. Para a Classe I de Vertucci, a radiografia mostrou maior sensibilidade (94,7%), especificidade (64,9%) e AUC (0,795) do que a radiovisiografia (89,3%, 62,2% e 0,757, respectivamente), assim como para a Classe V (Radiografia 69,2%, 93% e 0,811; Radiovisiografia 50%, 84,2% e 0,671, respectivamente). Nenhuma das técnicas contribuiu para o diagnóstico da Classe III (AUC <0,5). A ramificação foi pouco frequente (2,9%) e a detecção foi baixa (Sensibilidade 25% para radiografia e 0% para radiovisiografia).

**Discussão:**

Este é o primeiro estudo para avaliar a reprodutibilidade e validade dessas duas técnicas radiográficas em comparação com a TCFC para a detecção de variações anatômicas nos dentes.

**Conclusões:**

A radiografia com placas de fósforo apresentou maior reprodutibilidade e validade para o diagnóstico da Classe I e V de Vertucci, que foram as variações mais frequentes. Este foi um trabalho de conclusão de durso para o título de Mestre em Odontologia e estará no repositório da Universidad Santo Tomas seccional Bucaramanga.

## Introducción

En 1974, Vertucci definió que uno de los objetivos principales en el tratamiento endodóntico es la adecuación del sistema de conductos radiculares a través de una limpieza integral químio-mecánica, con el fin de eliminar tejidos orgánicos y bacterias y lograr un sellado tridimensional. Además, evidenció que 69% de los premolares presentaban un conducto, 24% tenían una configuración 1:2, 4% tenían una configuración 1:2:1, y 1% presentaba 2 conductos independientes, permitiendo considerar la existencia de variaciones anatómicas como bifurcaciones o conductos dobles en tratamientos endodónticos que inexplicablemente fracasan^1^. Ahmed y colaboradores demostraron que entre 15,8% y 21,5% de los primeros premolares inferiores presentan patologías pulpares y periapicales, y presentan alto índice de fracasos terapéuticos^2^. Por lo anterior, todos los procedimientos endodónticos requieren imágenes con alta resolución espacial para analizar adecuadamente la estructura de los conductos, su longitud y las lesiones presentes de origen endodóntico.

La tomografía computarizada de haz cónico (CBCT) se utiliza en odontología clínica para examinar los dientes y sus tejidos circundantes, y tiene identificación anatómica en tres dimensiones que permite la detección de lesiones periapicales y evaluación de la morfología del sistema de conductos radiculares, con una menor dosis de radiación efectiva que la tomografía tradicional; por lo cual es considerada estándar de oro para el diagnóstico y tratamiento de problemas endodónticos. Sin embargo, su capacidad para mostrar características anatómicas y patologías se afecta por la unidad de exploración, el campo de visión (FOV), el objeto examinado, el tiempo de exploración, el voltaje del tubo, el amperaje y el tamaño del vóxel^3^. Por ejemplo, Versiani y colaboradores mencionan algunas desventajas como una resolución espacial insuficiente y el grosor del corte que impide detectar configuraciones anatómicas más complejas, como las ramificaciones más finas, entre otras^4^^-^^5^. Además, la CBCT presenta limitaciones de acceso y costo, y los índices de radiación ionizante muchas veces no están totalmente calibrados cuando se usan equipos y técnicas no específicas para dientes únicos. Estas limitaciones deben ser consideradas cuando se selecciona este tipo de examen diagnóstico para procedimientos endodónticos^6^.

Las radiografías digitales (radiovisiografia) o digitalizadas (placa de fósforo) han sido utilizadas durante siglos para el diagnóstico en cada consultorio odontológico, especialmente en el servicio de endodoncia, y son una ayuda imprescindible en todas las fases del tratamiento endodóntico, pues cada paso requiere la verificación radiográfica. Con estas imágenes se puede visualizar en dos dimensiones el diente en su interior y los tejidos y estructuras anatómicas circundantes, de acuerdo con la posición en donde se ubique el cono del equipo de RX. Sin embargo, presentan desventajas como son su magnificación en un rango de 17% al 25% y la superposición de estructuras anatómicas lo que dificulta identificar fácilmente la anatomía real del conducto, entre otras^6^^-^^7^.

El clínico debe tener conocimiento de las características esenciales de la tecnología para seleccionar el equipo y la técnica que le ofrezca imágenes con mayor resolución y contraste y con mejor perfil de riesgo/beneficio^8^, que le permitan evidenciar las características del sistema de conductos radiculares que por su variación y tamaño son difíciles de observar con otros medios de diagnóstico clínico. Por tanto, es importante analizar diferentes técnicas estandarizadas efectivas *in vitro*^9^ para determinar qué clase de radiografía es la más confiable al compararla con la CBCT como estándar de oro. Por lo anterior, el objetivo fue establecer la reproducibilidadintra e inter observador de la lectura de la radiografía digitalizada con placas de fósforo y la radiovisiografía digital con sensor, as*í* como, la reproducibilidad inter-observador de la lectura de la CBCT y la validez de criterio de las primeras dos técnicas para identificar las variaciones anatómicas de primeros premolares inferiores comparadas con CBCT*.*

## Materiales y Métodos

Tipo de estudio y muestra: Este es un estudio de evaluación de tecnología diagnóstica, con una población conformada por los dientes caracterizados como primeros premolares inferiores con formación apical completa del Banco de Dientes Humanos Permanentes de la Universidad Santo Tomás de Floridablanca Santander, Colombia. Se excluyeron los dientes con restauraciones metálicas o con fracturas radiculares o coronales complicadas. El muestreo fue de corte transversal aplicando a la totalidad de la muestra las tres técnicas: la CBCT, la técnica de radiografías digitales con sensor y radiografías digitalizadas con placa de fósforo.

Procedimientos: Los dientes, que estaban conservados en un medio de formalina al 10%, fueron ubicados en una placa de acrílico que simuló la mandíbula, las tres pruebas se realizaron en un periodo de tiempo de 4 meses entre julio del 2020 y noviembre del mismo año. Para la toma de imágenes con CBCT se utilizó un Tomógrafo marca Plan Meca Promax 3D Classic con las siguientes características: vóxel 1,25 p, 90 Kv, 14 mA, pantalla plana, montado en un brazo en forma de U que se giró horizontalmente alrededor del modelo de dientes, con tiempo de exposición de 2-5 seg, la imagen capturada tenía un aumento promedio aproximado de 10% con respecto a las medidas reales, un tamaño de foco medio en el rayo central de 0,5 mm, y una distancia entre el foco y el modelo de 10 mm aproximadamente. El FOV utilizado fue de 8 X 5 cm^3^, se colocó el modelo en una unidad simulando posición del paciente y el equipo se programó para una rotación única de 360°. El modelo permaneció inmóvil durante la exposición^10^. Cada imagen de CBCT fue evaluada en una sola oportunidad por cada evaluador de forma independiente.

Para la adquisición de las imágenes radiográficas con sensor y con placa de fósforo se utilizó un equipo Rayos X Heliodent Plus de Denstplay Sirona. El sensor utilizado fue marca Schick con una dimensión externa estándar de 37 x 24 mm, con una zona activa de 30 x 20 mm, un grosor < 5 mm, una reducción según el fabricante de radiación de 80 - 90% y una vida útil de 400,000 dosis. Para la placa de fósforo se usó un equipo versión 1 Satelec Acteon/PS Pix. La tensión del tubo fue seleccionable a 70 kV, la corriente del tubo de 7 mA, con un foco de 0,4 (IEC 336) y un voltaje de 120-240 V ± 10% sin conmutación, 50-60 Hz y un tiempo de registro que fue ajustable de 0,01 s a 3,2 s^11^. Las radiografías fueron tomadas de manera Mesio radial con angulación de 35° de acuerdo con la técnica recomendada por Martínez Lozano^12^^-^^13^, garantizando una angulación horizontal de 20°- 40°, que permitió ver el número de conductos que estaban presentes (Figura). Además, esta técnica fue validada por Vertucci en 1984 para identificar la anatomía según su clasificación^14^. Las imágenes fueron procesadas en un software de radiología digital basado en el estándar internacional para Imagen & Comunicación Digital en Medicina conocido como CDR DICOM® cuando se utilizó el sensor. Las placas de fósforo fueron escaneadas con una resolución de 22 pl./mm. Las imágenes se transmitieron directamente al software de obtención de imágenes Sidexis 4 de un Scanner PS Pix Versión 1 Satelec Acteon. El tamaño utilizado fue de 2 de 31 x 41 mm.

Todas las imágenes obtenidas fueron evaluadas por dos endodoncistas previamente capacitados por un odontólogo especialista en radiología en los tres planos: sagital, axial (en dirección coronal a apical y de apical a coronal) y plano coronal, por desplazamientos en las imágenes a través de los diferentes planos. Los evaluadores identificaron la configuración de los conductos según clasificación de Vertucci 1984, en cada uno de los dientes, así como la presencia en cada una de las ramificaciones^14^. Cada imagen radiográfica con sensor y con placa de fósforo fue evaluada dos veces por cada evaluador, de forma independiente, para esto se aleatorizaron las imágenes, y las lecturas se realizaron con una diferencia de al menos 15 días.

Análisis de la información: Para cada técnica se determinó por separado la reproducibilidad intra e inter-evaluador utilizando el coeficiente Kappa de Cohen tanto para la configuración de los conductos como para la presencia de ramificación de los conductos. Para cada coeficiente Kappa se calculó el respectivo intervalo de confianza del 95% (IC95%). Posteriormente, se consideró como estándar de oro la clasificación de Vertucci por CBCT según la evaluación final concordante por los dos observadores, para cada configuración y para cada técnica radiográfica se calculó la sensibilidad y la especificidad. Esto se hizo clasificando cada diente de forma binaria (presencia o ausencia de cada Clase específica). Adicionalmente se evaluó la sensibilidad y la especificidad de cada técnica para la detección de ramificaciones. También se calcularon los Valores predictivos positivos y negativos (VPP y VPN) y el área bajo la curva ROC (AUC) con el IC95%. El análisis se realizó con el programa Stata 12.

Este estudio fue aprobado por el comité de ética institucional de la USTA.

## Resultados

### Reproducibilidad intra-observador de la lectura de las imágenes tomadas con placas de fósforo y con sensor

El primer evaluador, para las placas de fósforo, tuvo un acuerdo entre la primera y la segunda lectura para la clasificación de Vertucci de 96,4% con una reproducibilidad alta (Kappa de Cohen: 0,902; IC95% 0,797 - 0,976), sin embargo, aunque para las ramificaciones el acuerdo fue de 94,3%, la reproducibilidad fue baja (Kappa de Cohen: 0,172; IC95% -0,167 - 0,510). En cuanto a las imágenes tomadas con radiovisiografía con sensor, el acuerdo obtenido fue del 91,4% con una reproducibilidad buena para la clasificación de Vertucci (Kappa de Cohen: 0,774; IC95% 0,654 - 0,871) mientras que en las ramificaciones el acuerdo fue del 90% y la reproducibilidad fue baja (Kappa de Cohen: 0,311; IC95% 0,042 - 0,580).

El segundo evaluador, para placas de fósforo, tuvo un cuerdo del 98,6% y una reproducibilidad alta (Kappa de Cohen: 0,975; IC95% 0,928 - 0,977), y para las ramificaciones el acuerdo fue de 100% y la reproducibilidad fue excelente (Kappa de Cohen: 1,0; IC95% 1,0 - 1,0). Para las imágenes de radiovisiografía con sensor su acuerdo fue del 97,1%, con una reproducibilidad alta (Kappa de Cohen: 0,954; IC95% 0,909 - 0,989); de la misma forma para las ramificaciones el acuerdo fue del 94,3% y la reproducibilidad fue alta (Kappa de Cohen: 0,860; IC95% 0,767 - 0,954).

### Reproducibilidad inter-observador de la lectura de las imágenes tomadas con CBCT, con placas de fósforo y con sensor

Para la clasificación de Vertucci, el acuerdo en las lecturas de las imágenes CBCT entre los dos observadores fue del 93,5% con una reproducibilidad alta (Kappa de Cohen: 0,856; IC95% 0,759 - 0,936) y para las ramificaciones el acuerdo fue de 98,6% con una reproducibilidad buena (Kappa de Cohen: 0,793; IC95% 0,514 - 1,000). En cuanto a las imágenes de placas de fósforo para la clasificación de Vertucci el acuerdo fue de 49,3% con una reproducibilidad baja (Kappa de Cohen: 0,202; IC95% 0,125 - 0,293) y aunque para ramificaciones el acuerdo fue de 92,9%, la reproducibilidad fue baja (Kappa de Cohen de 0,129; IC95% -0,164 - 0,422). La reproducibilidad inter-observador de las lecturas de las imágenes tomadas con radiovisiografía con sensor fue más baja que las observadas para las placas de fósforo, tanto para la clasificación de Vertucci (acuerdo de 41,4% y Kappa de Cohen 0,139; IC95% 0,071 - 0,229) como para las ramificaciones (acuerdo de 72,9% y Kappa de Cohen 0,126; IC95% -0,030 - 0,282).

### Sensibilidad, especificidad, VPP, VPN y AUC de la radiografía con placas de fósforo y la radiovisiografía con sensor comparadas con la CBCT como estándar de oro

Considerando el diagnóstico realizado por los dos observadores, o tres cuando hubo desacuerdo (10,7%), con las imágenes de la CBCT se realizó la caracterización anatómica de los primeros premolares inferiores estudiados. Se encontró que el 73,6% fueron Clase I de la clasificación de Vertucci, 5% Clase III, 18,6% Clase V, 0,7% Clase VIII y 2,1% sin Clasificación o Clase 0. En cuanto a las ramificaciones, estuvieron presentes en un 2,9% de los dientes.

El 94,2% de los dientes clasificados por la CBCT como Clase I fueron también identificados por la placa de fósforo, mientras que ninguno de los dientes clase III fueron identificados por esta técnica y el 69,2% de la clase IV fueron reconocidos como tal (Tabla 1). Por otra parte, la radiovisiografía permitió diagnosticar el 89,3% de los dientes Clase I y el 50% de los dientes Clase V; similar a lo observado previamente no se identificó ningún diente como Clase III con esta técnica (Tabla 2). Para evaluar la validez de criterio de la radiografía con placas de fósforo y la radiovisiografía con sensor respecto a la CBCT se utilizaron las tres Clases de configuración de Vertucci más frecuentes. Para la Clase I y V, la placa de fósforo tuvo mayor sensibilidad, especificidad y AUC que la radiovisografía. Sin embargo, para la Clase III la sensibilidad de las dos técnicas fue de 0% y las dos técnicas tuvieron una AUC inferior a 0,5, es decir que ninguna de las dos técnicas aporta para el diagnóstico de esta Clase (Tabla 3).

Para la detección de ramificaciones, con la imagen tomada con placa de fósforo se pudo identificar uno de los cuatro dientes con esta variación anatómica, mientras que la radiviosiografía no detectó ninguno de estos dientes (Tabla 4), teniendo las dos técnicas una baja sensibilidad, con una AUC que indica que la primera técnica tiene baja discriminación, y que la segunda técnica no aporta al diagnóstico de la presencia de ramificaciones en los primeros premolares inferiores (Tabla 3).

**Tabla 1:** Caracterización anatómica según Vertucci de los primeros premolares inferiores con placas de fósforo comparada con CBCT (N=140)

Prueba			CBCT		
Fósforo	Clase I n (%)	Clase III n (%)	Clase V n (%)	Clase VII n (%)	Clase 0 n (%)
Clase I	97 (94,2)	6 (86,7)	7 (26,9)	0	0
Clase III	2 (1,9)	0	1 (3,9)	1 (100)	0
Clase V	4 (3,9)	1 (14,3)	18 (69,2)	0	3 (100)
Clase VII	0	0	0	0	0
Clase 0	0	0	0	0	0
Total	103	7	26	1	3

**Tabla 2:** Caracterización anatómica según Vertucci de los primeros premolares inferiores con radiovisiografía comparada con CBCT (N=140)

Prueba	CBCT				
Radiovisiografía	Clase I n (%)	Clase III n (%)	Clase V n (%)	Clase VII n (%)	Clase 0 n (%)
Clase I	92 (89,3)	3 (42,9)	11 (42,3)	0	0
Clase III	1 (1)	0	1 (3,8)	0	0
Clase V	10 (9,7)	4 (57,1)	13(50)	1 (100)	3 (100)
Clase VII	0	0	0	0	0
Clase 0	0	0	1 (3,8)	0	0
Tipo 0	0	0	1 (3,8)	0	0
Total	103	7	26	1	3

**Tabla 3:** Caracteríticas operativas de las técnicas con placa de fósforo y con radiovisiografía para la detección de las Clases I, III y V de Vertucci, y las ramificaciones en los primeros premolares inferiores utilizando como estándar de oro la CBCT.

Prueba	Sensibilidad	Especificidad	VPP	VPN	AUC (IC95%)	
Vertucci I	%	%	%	%		
Placa de fósforo	94,7	64,9	88,2	80	0,795 (0,714	- 0,876)
Radiovisiografía	89,3	62,2	86,8	67,6	0,757 (0,673	- 0,842)
Vertucci III						
Placa de fósforo	0	97%	0	94,9	0,485 (0,470	- 0,500)
Radiovisiografía	0	98,5%	0	94,9	0,493 (0,482	- 0,503)
Vertucci V						
Placa de fósforo	69,2	93	69,2	93	0,811 (0,718	- 0,905)
Radiovisiografía	50	84,2	41,9	88,1	0,671 ( 0,568	- 0,775)
Ramificaciones						
Placa de fósforo	25	96,3	16,7	97,8	0,607 (0,361	- 0,852)
Radiovisiografía	0	90,4	0	96,9	0,452 (0,427	- 0,477)

**Tabla 4:** Detección de las ramificaciones con placa de fósforo y con radiovisiografía com paradas con CBCT en primeros premolares inferiores (N=140)

Prueba	CBCT		Total
Placa de fósforo	Si	No	
Si	1	5	6
No	3	131	134
Radiovisiografía			
Si	0	13	13
No	4	123	125
Total	4	136	140

## Discusión

Al evaluar las imágenes adquiridas con CBCT se evidenció predominancia de la configuración Clase I de Vertucci; seguida por la Clase V y la Clase III, similar a lo reportado por Vertucci en 1974 mediante técnica de clarificación, donde se evidenció la configuración Clase I en 69% de los dientes, Clase V en 24%, Clase III en 4% y Clase IV en 1%^1^. De igual manera estos resultados son equivalentes a los del estudio de Zillich y colaboradores, quienes reportaron 66% de Clase ^15^I, así como los de Velmorugen y colaboradores quienes reportaron un 72% de Clase I, 3% de III y 8% de V en población hindú^16^, y los de Rodríguez y colaboradores quienes reportaron 67,1% de Clase I y 21% de V en población colombiana^17^. Así mismo, Zhang y colaboradores utilizando CBCT, identificaron en un 74,1% Clase I, 14% Clase V y 3,5% Clase III en población China^18^. Adicionalmente, en el presente estudio se determinó sin Clasificación a 2,1% de los premolares inferiores, esta frecuencia es menor a la reportada en población Birmana (7,69%)^19^y en población China (7,7%)^20^.

En cuanto a las ramificaciones, se detectaron con la CBCT en un 2,9% de los premolares inferiores, estos resultados son similares a los reportados por Zillich y colaboradores en premolares inferiores (1,5%)^15^, pero difieren de los reportados por Riccuci y Siqueira, quienes evidenciaron ramificaciones en molares y premolares superiores hasta en un 80% con CBCT y Microtomografías^21^, por Pineda y colaboradores quienes las reportaron en un 80% en el conducto principal de premolares inferiores ubicados en tercio apical^22^, y por Caliskan y colaboradores quienes encontraron ramificaciones en premolares inferiores, ubicados en el tercio medio en un 52% de los casos y deltas apicales en un 21,6%^23^.

Al respecto de la reproducibilidad intra-observador esta varió, siendo menor para el primer evaluador especialmente para la presencia de ramificaciones con las dos técnicas radiográficas, esto podría deberse a la diferencia en años de experiencia como Endodoncistas entre los dos observadores, puesto que el segundo, que tiene cerca de diez años más de experiencia, tuvo una reproducibilidad alta (Kappa de Cohen >0,850) tanto para la clasificación de Vertucci como para la visualización de imágenes sugestivas de ramificaciones en las placas de fósforo y en las radiovisiografía con sensor. Esto debe tenerse en cuenta en el momento de reconfirmar un diagnóstico en diferentes tiempos por un mismo observador especializado.

En cuanto a la reproducibilidad inter-observador, la CBCT tuvo una alta reproducibilidad para la clasificación de Vertucci y buena reproducibilidad para las ramificaciones. Sin embargo, la reproducibilidad inter-observador, tanto para Vertucci como para las ramificaciones, fue baja para las dos técnicas, siendo menos favorable para la radiovisiografía con sensor. Lo anterior soporta la hipótesis de que, además del entrenamiento y la experiencia del profesional, estas técnicas radiológicas revisten mayor dificultad para observar la anatomía del primer premolar inferior. Aunque en la literatura revisada no se encontraron estudios que evalúen la reproducibilidad de estas dos técnicas para detectar las variaciones anatómicas, otros autores han reportado resultados similares de mayor reproducibilidad inter-observador para la CBCT que para la radiovisografía y la radiografía con placa de fósforo para la detección de accidentes periodontales como dehiscencias y fenestración ósea^24^^,^^25^.

Al analizar la validez de la placa de fósforo y la radiovisiografía comparadas con la CBCT para la configuración de Vertucci, se obtuvo una alta sensibilidad para el diagnóstico de la Clase I con las dos técnicas, siendo la sensibilidad de la primera técnica mayor (94,7% y 89,3%, respectivamente). Aunque la especificidad de las técnicas fue menor para esta Clase (64,9% y 62,2%, respectivamente), las AUC indican que ambas técnicas aportan en la discriminación de esta variación anatómica en los primeros premolares inferiores. Para la Clase V se observó un comportamiento diferente, siendo mayor la especificidad que la sensibilidad de las dos técnicas, sin embargo, el AUC de la placa de fósforo fue mayor. Por otra parte, para la Clase III se observó que ninguna de las técnicas aporta en el diagnóstico de esta (AUC <0,5). Estas técnicas tampoco tuvieron buen rendimiento para la detección de ramificaciones puesto que la sensibilidad fue muy baja. Teniendo en cuenta los resultados de sensibilidad, especificidad, valores predictivos y AUC, que el 92% de los primeros premolares inferiores tuvieron una clasificación de Vertucci I o V, y que solamente cerca del 3% presentaron ramificaciones, se podría recomendar que en la evaluación de diagnóstico endodóntico se utilice la radiografía digitalizadas con placa de fósforo. Además, en el caso de presentarse fracaso en el tratamiento se recomendaría recurrir a la CBCT, considerando que la causa del fracaso puede ser la presencia de otra clasificación anatómica o de ramificaciones no evidenciadas inicialmente. Sin embargo, en otros contextos donde las variaciones I y V no sean las más frecuentes o que la presencia de ramificaciones sea más frecuente se recomendaría utilizar como primera opción diagnóstica la CBCT o realizar estudios de validación de estas técnicas.

De acuerdo con la revisión realizada este es el primer estudio que evalúa la validez de estas técnicas para la detección de variaciones anatómicas en el primer premolar inferior, por tanto, no se pueden discutir estos hallazgos particularmente. Sin embargo, otros autores que compararon estas técnicas con la CBCT para detectar perforaciones en raíces reportaron un buen rendimiento de las dos (radiovisiografía con sensor: sensibilidad de 81,3% y especificidad de 93,8%; radiografías digitalizadas con placas de fósforo: sensibilidad de 85,4% y especificidad de 91,7%)^26^Adicionalmente, en un meta análisis se reportó un AUC de 0,72 para discriminación de la periodontitis apical por la radiovisiografía comparada con la CBCT^27^.

Dentro de las limitaciones del presente estudio se encuentran el hecho de que no se contó con información de la edad, el sexo ni la procedencia de los dientes lo que dificultó valorar el riesgo de sesgo de selección. Además, el tamaño de muestra limitó la precisión de las determinaciones evidenciándose en los intervalos de confianza amplios, y limitó la evaluación de la validez a las tres clasificaciones de Vertucci más frecuentes. Al respecto, las Clases VII y 0, aunque infrecuentes, no fueron detectadas por las dos técnicas (Tabla 2 y 3), este tipo de variaciones anatómicas se deben tener en cuenta para el desarrollo de tratamientos de conductos, puesto que complejizan el pronóstico de la endodoncia cuando no se detectan por las tecnologías diagnósticas utilizadas.

## Conclusiones

En los primeros premolares inferiores de la población estudiada, las variaciones anatómicas Clase I y V de Vertucci fueron las más frecuentes, similar a lo reportado en la literatura internacional, mientras que las ramificaciones fueron infrecuentes. La reproducibilidad intra- evaluador varió siendo menor para la presencia de ramificaciones. La reproducibilidad inter evaluador fue mayor cuando se utilizó la CBCT, tanto para la clasificación de Vertucci como para las ramificaciones, al compararse con la radiovisiografía con sensor y la radiografía con placa de fósforo. Finalmente, el rendimiento diagnóstico de la radiografía con placa de fósforo fue mayor al de la radiovisiografía con sensor para la detección de las variaciones anatómicas Clase I y V de Vertucci teniendo como estándar de oro la CBCT.
